# Urinary and plasma donor-derived cell-free DNA for noninvasive monitoring of BK polyomavirus-associated nephropathy in kidney transplant recipients: a prospective cohort study

**DOI:** 10.1080/0886022X.2025.2521452

**Published:** 2025-06-25

**Authors:** Luying Guo, Sulin Luo, Rongfang Shen, Pengpeng Yan, Meifang Wang, Tianlu Zhang, Junhao Lv, Guangjun Liu, Hongfeng Huang, Zhimin Chen, Huiping Wang, Wenhan Peng, Jianyong Wu, Jianghua Chen, Rending Wang

**Affiliations:** ^a^ Kidney Disease Center, the First Affiliated Hospital, School of Medicine, Zhejiang University, Hangzhou, Zhejiang Province, China; ^b^ Key Laboratory of Kidney Disease Prevention and Control Technology, Hangzhou, Zhejiang Province, China; ^c^ National Key Clinical Department of Kidney Diseases, Hangzhou, Zhejiang Province, China; ^d^ Institute of Nephrology, Zhejiang University, Hangzhou, Zhejiang Province, China; ^e^ Zhejiang Clinical Research Center of Kidney and Urinary System Disease, Hangzhou, Zhejiang Province, China; ^f^ Affiliated Hospital of Shaoxing University, Shaoxing, Zhejiang Province, China

**Keywords:** BKPyVAN, kidney transplantation, donor-derived cell-free DNA, allograft rejection, dynamic surveillance

## Abstract

**Background:**

BK polyomavirus-associated nephropathy (BKPyVAN) is a major cause of allograft injury and dysfunction in kidney transplant recipients. Current monitoring tools, including viremia and biopsy, have limitations in sensitivity, invasiveness, and timing.

**Objective:**

To evaluate donor-derived cell-free DNA (dd-cfDNA) in urine and plasma as a dynamic, noninvasive biomarker for monitoring treatment response and predicting rejection risk in patients with biopsy-proven BKPyVAN.

**Methods:**

In this prospective cohort study, 25 kidney transplant recipients with biopsy-proved BKPyVAN were enrolled and stratified into two cohorts: conventional immunosuppression reduction (CISR, *n* = 20) and early immunosuppression reduction (EISR, *n* = 5). A total of 224 urine and plasma samples were collected before biopsy and at 1, 2, 3, and 6 months post-biopsy. dd-cfDNA levels were quantified and correlated with histological features and clinical outcomes.

**Results:**

Urinary dd-cfDNA levels significantly declined in the CISR cohort by month 2 (*p* < 0.01), preceding changes in creatinine and BKPyV reads. In the EISR cohort, urinary dd-cfDNA levels remained stable, suggesting early therapeutic response. Plasma dd-cfDNA effectively identified acute rejection, with elevations in two CISR patients. Histologic injury patterns, including edema and cast formation, correlated with urinary dd-cfDNA concentrations (*r* = 0.44–0.51, *p* < 0.05).

**Conclusion:**

Combined urinary and plasma dd-cfDNA measurements are promising for noninvasive, dynamic surveillance of BKPyVAN and rejection risk in kidney transplant recipients. Larger, multicenter studies are warranted to define clinical thresholds and standardize integration into immunosuppression management.

## GRAPHICAL ABSTRACT

**Figure:**
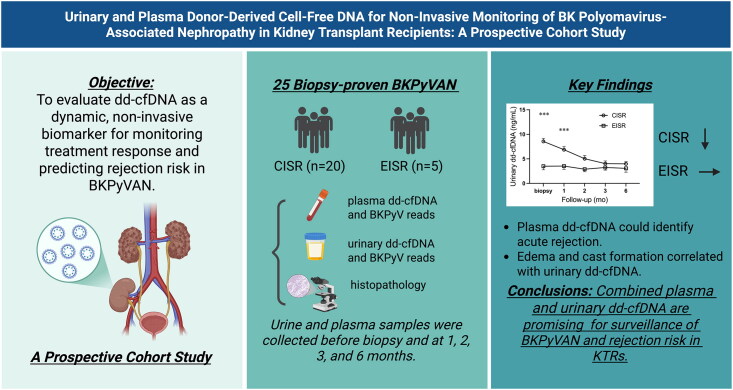

## Background

BK polyomavirus-associated nephropathy (BKPyVAN) is one of the leading causes of graft injury and loss in patients with kidney transplantation [1]. The mainstay of treatment for BKPyVAN is the reduction of immunosuppression with the primary goal of maintaining stable allograft function. Due to insufficient immunosuppression, BKPyVAN is associated with an increased risk of allograft rejection [2–4]. Accordingly, monitoring the allograft health status is of particular importance. Urinary and plasma BK polyomavirus (BKPyV) loads are routinely measured in BKPyVAN surveillance. However, BKPyV viremia can persist for over a year in BKPyVAN [5]. Repeated allograft biopsy remains the gold standard for demonstrating disease remission and early signs of rejection. Nevertheless, positive staining for simian virus 40 (SV40) can persist for approximately 5 months, which can lead to increased tubular atrophy and interstitial fibrosis over time [5]. Therefore, reducing immunosuppression based solely on positive SV40 tissue staining could increase the risk of rejection. Additionally, the invasive nature of biopsies makes them less suitable for dynamic monitoring. Presently, there is a crucial need for a sensitive and noninvasive method for BKPyVAN monitoring.

Donor-derived cell-free DNA (dd-cfDNA) is an emerging noninvasive biomarker for monitoring allograft status [6]. Its potential as an alternative to allograft biopsies has been increasingly recognized [7]. Due to its relatively short half-life (approximately 5 to 150 min), dd-cfDNA levels can provide real-time insights into the allograft status. Plasma dd-cfDNA levels exhibit superior sensitivity and specificity in detecting allograft injuries, especially in antibody-mediated rejection, compared to conventional indicators such as serum creatinine and cytokines, including CXCL9 and CXCL10 [8,9]. In our previous study, a reduction in plasma dd-cfDNA level was a more sensitive indicator of early response to anti-rejection therapy compared to serum creatinine levels [10]. Extensive explorations of plasma dd-cfDNA in BKPyVAN have been conducted in the context of kidney transplantation [9,11]. However, a consensus on its distinctive characteristics has not yet been reached.

In addition to circulating dd-cfDNA, successful extraction of cell-free DNA (cfDNA) from urine has enabled the identification of urinary dd-cfDNA to demonstrate graft injury [12]. Recent researches, including our own, have demonstrated the clinical significance of urinary dd-cfDNA levels in BKPyVAN encompassing applications include diagnosing disease [13], distinguishing between BKPyVAN and T cell-mediated rejection (TCMR) [14], and assessing disease progression [15]. These findings highlight the potential utility of urinary and plasma dd-cfDNA in dynamic surveillance during BKPyVAN treatment, outperforming traditional markers such as creatinine levels and BKPyV loads to effectively characterize disease remission and identify rejection risk. Despite the growing interest in urinary and plasma dd-cfDNA detection, its correlation with allograft injury in BKPyVAN and clinical significance in guiding management adjustments remain unclear.

In this study, we initiated a prospective evaluation of the dynamics of urinary and plasma dd-cfDNA levels during BKPyVAN treatment, their association with allograft injury, and their potential to identify the risk of acute rejection.

## Methods

### Study design and patients

**Figure 1.**
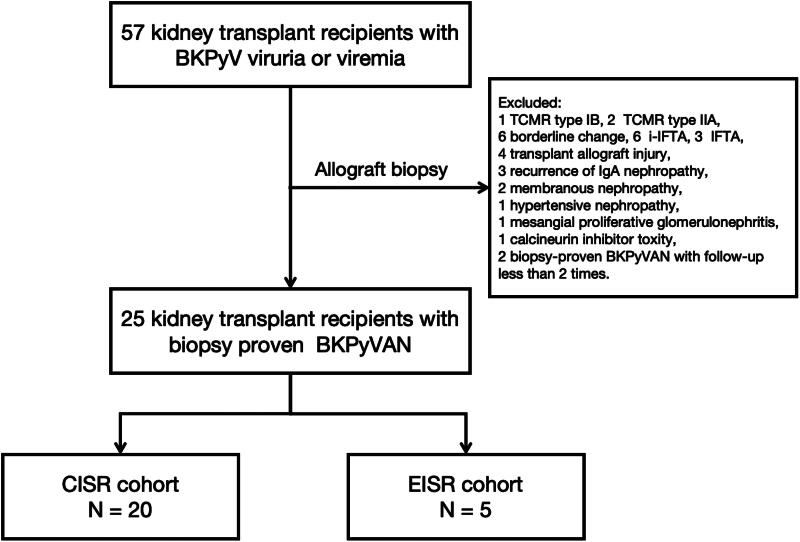
Flowchart of the study. Among the 57 patients enrolled, 32 cases were excluded, including one patient with TCMR type IB, two with TCMR type IIA, six with borderline changes, six with i-IFTA, three with IFTA, four with transplant allograft injury, three with recurrence of IgA nephropathy, two with renal allograft membranous nephropathy, one with post-transplant hypertensive nephropathy, one with allograft mesangial proliferative glomerulonephritis, one with calcineurin inhibitor toxicity, and two biopsy-proven BKPyVAN patients with fewer than two follow-up visits. The 25 kidney transplant recipients with biopsy-proven BKPyVAN were further divided into 20 patients with conventional immunosuppression (CISR cohort) reduction and 5 with early immunosuppression reduction before biopsy (EISR cohort).

A total of 57 adult kidney transplant recipients with persistently elevated urinary BKPyV loads (>7 log10 c/mL) and BKPyV-DNAemia (>3 log10 c/mL) sustained for more than 3 weeks [16] were assessed for eligibility in a prospective, case-based, observational, single-center study. The study aimed to examine the clinical value of both plasma and urinary dd-cfDNA levels for BKPyVAN monitoring. This study was approved by the Research Ethics Committee of the First Affiliated Hospital, School of Medicine, Zhejiang University (Hangzhou, China; Approval Number 2017-642-1) and was conducted according to the principles of the Declaration of Helsinki (2000). The patients underwent allograft biopsies to reveal histopathology. Among the 57 patients enrolled, 25 with biopsy-proven BKPyVAN were included in subsequent investigations. The remaining cases were excluded, including one patient with TCMR type IB, two with TCMR type IIA, six with borderline changes, six with i-IFTA, three with IFTA, four with transplant allograft injury, three with recurrence of IgA nephropathy, two with renal allograft membranous nephropathy, one with post-transplant hypertensive nephropathy, one with allograft mesangial proliferative glomerulonephritis, one with calcineurin inhibitor toxicity, and two biopsy-proven BKPyVAN patients with fewer than two follow-up visits. Peripheral blood and urine samples from the enrolled patients were collected within 24 h before the biopsy and at 1, 2, 3, and 6 months afterwards. Twenty-five biopsy-proven BKPyVAN patients with 224 samples (112 urine and 112 plasma samples) were included in this study (Flowchart, Figure 1). Written informed consent was obtained from all patients before the study commenced.

### Definition of BKPyVAN diagnosis

Kidney transplant recipients exhibiting elevated serum creatinine levels along with persistent BKPyV viruria or viremia were suspected to have developed BKPyVAN. Subsequently, these individuals received an allograft biopsy with indication. BKPyVAN diagnosis was confirmed by two experienced pathologists based on histological changes observed in the biopsy, including positive SV40 staining, interstitial infiltrates, tubular injury, and tubulitis according to the Banff Polyomavirus Nephropathy classification system [17].

### Measurement of dd-cfDNA and BKPyV DNA reads

Conventional midstream clean-catch urine and peripheral blood samples were collected into cfDNA collecting tubes within 24 h before allograft biopsy, 1, 2, 3, and 6 months during follow-up. The samples were stored at 4–37 °C for less than 24 h before centrifugation. Subsequently, the samples underwent processing, cfDNA extraction, and preservation. The levels of dd-cfDNA were measured through library construction, target region capture sequencing, and quantification based on our established protocol [14]. We applied Kraken (Johns Hopkins University, Baltimore, MD, United States) as the taxonomy classifier by mapping the k-mers of the query sequence to the lowest common ancestor of reference genomes. According to the algorithm of Kraken, sensitivity, and accuracy highly depend on database size. Therefore, we built a custom database that included BKPyV and JC polyomavirus (JCV). The SAMtools (http://samtools.sourceforge.net/) was employed to calculate the depth of the BKPyV genome sequence and count the number of BK reads under homogenization.

### BKPyVAN treatment and disease remission

The treatment commenced immediately upon clinical suspicion or biopsy confirmation of BKPyVAN, adjusted by urinary dd-cfDNA concentrations (>7.81 ng/mL) [14]. The most common changes in immunosuppressant regimens for BKPyVAN patients included converting mycophenolate to leflunomide and tacrolimus to ciclosporin, followed by reductions in tacrolimus and mycophenolate doses. Two patients were initiated with a mammalian target of rapamycin (mTOR) inhibitor. Additionally, five patients underwent prior immunosuppression reduction (6–10 weeks) before allograft biopsy (Table 1). Experienced clinicians identified disease remission based on a combination of urinary and plasma dd-cfDNA levels, urinary and plasma BKPyV loads, and serum creatinine levels. A reduction in urinary dd-cfDNA concentration to less than 4.4 ng/mL was considered indicative of disease remission [14].

**Table 1.** Immunosuppression changes after BKPyVAN.

Therapy	Numbers
EISR cohort (*n* = 5)	
Preceding immunosuppression reduction	5
CISR cohort (*n* = 20)	
Tacrolimus stopped	20
Mycophenolate reduced	2
Mycophenolate stopped	18
Cyclosporin started	20
mTOR started	2
Leflunomide started	18

CISR, conventional immunosuppression reduction; EISR, early immunosuppression reduction before biopsy; mTOR, mammalian target of rapamycin.

### Statistics

All statistical analyses in our study were conducted using GraphPad Prism version 9. Patient demographics, serum creatinine, and outcomes among subgroups were analyzed using the Mann-Whitney test (for continuous data) and the Chi-square test (for binary and ordinal data). The Kruskal-Wallis rank-sum test was used to assess longitudinal changes in urinary and plasma dd-cfDNA levels, followed by Dunn’s *post hoc* test to adjust for multiple comparisons. Pearson’s correlation analysis and linear regression were performed to evaluate the relationship between histopathological changes and dd-cfDNA. The resulting data were visualized using GraphPad Prism version 9. Statistical significance was defined as *p* values <0.05.

## Results

### Patient demographics

The research flowchart is illustrated in Figure 1. A total of 25 kidney transplant recipients with biopsy-proven BKPyVAN were enrolled for analysis in our study. According to the timing of reduced immunosuppression initiation, the patients were further divided into 20 with conventional immunosuppression reduction (CISR cohort) and 5 with early immunosuppression reduction before biopsy (EISR cohort). Their baseline characteristics are summarized in Table 2. The mean age of these BKPyVAN recipients was 45.64 ± 2.41 years, and donation after cardiac death (DCD) was predominant in these patients. Three patients (12%) underwent acute rejection in the CISR cohort. The median duration of biopsy-proven BKPyVAN was nearly half one year (185.5, IQR 138.8–354.8 days) after kidney transplantation. Recipient age, sex, primary kidney diseases, donor age and biologic sex, HLA-miss match, delayed graft function rate and immunosuppression induction were comparable between CISR and EISR cohorts. The baseline creatinine levels at biopsy-proven BKPyVAN were 192.0 ± 13.44 μmol/L for the total cohort, 196.5 ± 16.36 μmol/L for the CISR cohort, and 173.8 ± 15.15 μmol/L for the EISR cohort. The median urinary BKPyV loads were 3.45 × 10^8^ (6.43 × 10^6^–3.53 × 10^9^) copies/mL. In the EISR cohort, the urinary BKPyV loads were significantly lower than in the CISR cohort (1.30 × 10^7^, IQR: 2.0 × 10^6^–1.7 × 10^8^ copies/mL versus 4.1 × 10^8^, IQR: 9.5 × 10^6^–4.1 × 10^9^ copies/mL, *p* < 0.001). Next-generation sequencing was used to detect genome variants of BKPyV. The most common variants were BKPyV-IVc-1 and Ic, accounting for 36% and 32% of cases, respectively. The BKPyV-Ib, IVa-2, and IVa-3 were also commonly observed in our patients (Table 3 and Supplementary Figure 1).

**Table 2.** Patient demographics.

Characteristic	Total (*N* = 25)	CIRS (*N* = 20)	EIRS (*N* = 5)	*p* value
Age (years)	45.64 ± 2.41	46.40 ± 2.91	42.60 ± 3.33	0.287
Sex (M/F)	17/8	15/5	2/3	0.134
Primary kidney disease				0.458
Glomerulitis	20	15	5	
IgA nephropathy	3	3	0	
Others	2	2	0	
Donor age (years)	52.93 ± 2.47	51.73 ± 2.71	56.25 ± 5.87	0.458
Donor sex (M/F)	18/7	16/4	2/3	0.075
Donor source (DCD/LD)	21/4	18/2	3/2	0.102
HLA-MM	2.96 ± 0.39	3.06 ± 0.37	2.50 ± 1.50	0.063
Induction				0.307
Basiliximab	15	11	4	0.252
ATG	10	9	1	
DGF	5/20	3/17	2/3	0.252
Creatinine (μmol/L)	192.0 ± 13.44	196.5 ± 16.36	173.8 ± 15.15	0.146
Urinary BKPyV loads (copies/mL)	3.45 × 10^8^ (6.43 × 10^6^–3.53 × 10^9^)	4.1 × 10^8^ (9.5 × 10^6^–4.1 × 10^9^)	1.30 × 10^7^ (2.0 × 10^6^–1.7 × 10^8^)	<0.001
Duration of follow-up (days)	185.5 (138.8–354.8)	214.0 (144.0–373.0)	154.0 (105.5–894.0)	0.494
Acute rejection event	3	3	0	0.356

DCD, donation after cardiac death; LD, living donor; HLA-MM, HLA-mismatch; ATG; anti-thymocyte globulin; DGF, delayed graft function; CISR, conventional immunosuppression reduction; EISR, early immunosuppression reduction before biopsy.

**Table 3.** BK genome variants.

	BKV subtypes	Proportion
I	Ib-1	3
	Ic	8
II		0
III		0
IV	IVa-1	1
	IVa-2	2
	IVc-1	9
Undetectable		2

### Dynamics of urinary and plasma levels of dd-cfDNA during immunosuppression reduction therapy in BKPyVAN

**Figure 2.**
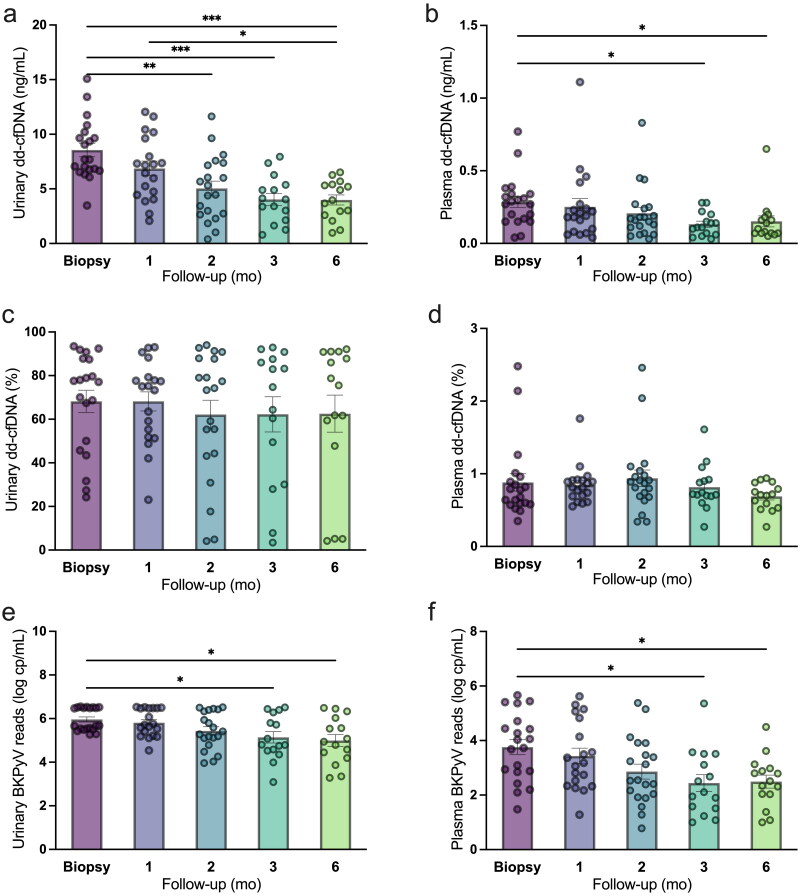
Dynamic surveillance of urinary and plasma dd-cfDNA and BKPyV reads in BKPyVAN patients with immediate reduced immunosuppression therapy at biopsy, 1, 2, 3, and 6 months afterwards. (a) urinary dd-cfDNA concentrations (ng/mL) were reduced at 1 month after BKPyVAN treatment, (b) plasma dd-cfDNA concentrations (ng/mL) were reduced at 3 months after BKPyVAN treatment, (c, d) urinary (%) and plasma (%) dd-cfDNA fractions were comparable during follow-up period, (e, f) urinary and plasma BKPyV reads (log copies/mL) declined 3 months afterwards. dd-cfDNA, donor-derived cell-free DNA; mean ± SE, *p < 0.05; **p < 0.01; ***p < 0.001.

In the CISR cohort, high levels of urinary dd-cfDNA concentrations were observed at allograft biopsy and the first month after immunosuppression reduction treatment in BKPyVAN with median levels of 7.70 (IQR: 6.69–10.06) and 7.16 (IQR: 4.45–9.62) ng/mL (Figure 2a). Subsequently, a decline in urinary dd-cfDNA concentrations was observed at 2, 3, and 6 months afterward, with median levels at 4.88, 4.04, and 3.96 ng/mL, respectively, which were significantly lower than baseline levels (all *p* < 0.01). Among the 20 patients in the CISR cohort, 18 individuals (90%) exhibited a reduction in urinary dd-cfDNA concentration by the second month. Plasma dd-cfDNA concentrations were comparable during the dynamic surveillance period with a decline tendency, which reached a statistical significance 3 months post-therapy (all *p* < 0.05, Figure 2b). Both urinary and plasma dd-cfDNA fractions were comparable among dynamic surveillance periods (Figures 2c-d). Similar to plasma dd-cfDNA concentrations, urinary and plasma BKPyV reads at 3 and 6 months after therapy were also significantly lower than baseline levels (all *p* < 0.05, Figures 2e-f). These results demonstrated that after 2 months of reduced immunosuppression therapy, the urinary dd-cfDNA concentration served as an earlier biomarker for reflecting BKPyVAN remission compared to other indicators.

### Comparison of dd-cfDNA dynamics between CISR and EISR cohorts in BKPyVAN

**Figure 3.**
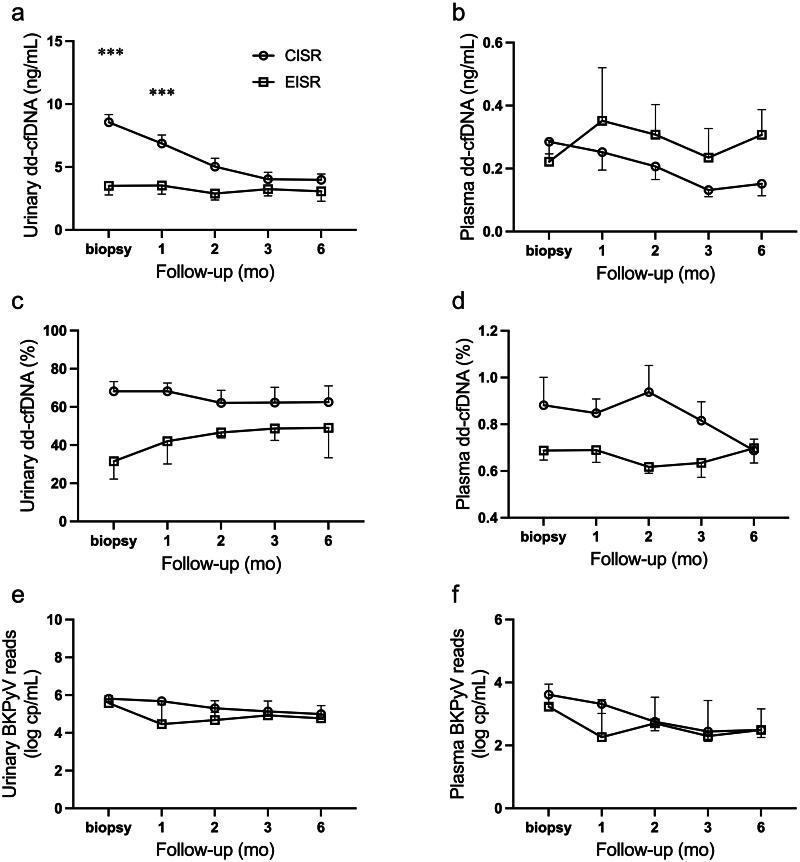
Comparison of urinary and plasma dd-cfDNA and BKPyV reads between CISR and EISR cohort. (a) Urinary dd-cfDNA concentrations were significantly higher in CISR cohort compared with EISR cohort at biopsy time and 1 month after BKPyVAN treatment (ng/mL). (b) plasma dd-cfDNA concentrations (ng/mL), (c, d) urinary and plasma dd-cfDNA fractions (%), (e, f) urinary and plasma BKPyV reads (log copies/mL) were comparable between CISR and EISR cohorts. dd-cfDNA, donor-derived cell-free DNA; mean ± SE, ***p < 0.001.

Five patients received preemptive immunosuppression reduction therapy (EISR cohort) upon clinical suspicion of BKPyVAN before allograft biopsy. Despite positive SV40 staining confirmed the diagnosis of BKPyVAN, the median urinary dd-cfDNA concentration at allograft biopsy was 3.69 ng/mL (IQR: 2.12–4.79) in the EISR cohort, leading to the maintenance of their current immunosuppression regimen. Dynamic surveillance revealed that urinary and plasma dd-cfDNA levels in the EISR cohort remained mostly stable within 6 months after biopsy (Figures 3a–d). In the CISR cohort, urinary dd-cfDNA concentrations were significantly higher 2 months after therapy initiation compared to the EISR cohort (both *p* < 0.001) but subsequently reached similar levels (Figure 3a). Both urinary and plasma BKPyV reads were comparable between the two cohorts (Figures 3e-f). Although serum creatinine and eGFR levels were similar in the CISR and EISR cohorts at biopsy, and at 1, 2, 3, and 6 months, decreased serum creatinine levels and increased eGFR levels were observed at 3 months in both cohorts (Supplementary Figure 2).

### Significance of dynamic urinary and plasma dd-cfDNA surveillance in BKPyVAN treatment, clinical decision making on withdrawal of immunosuppression reduction

**Figure 4.**
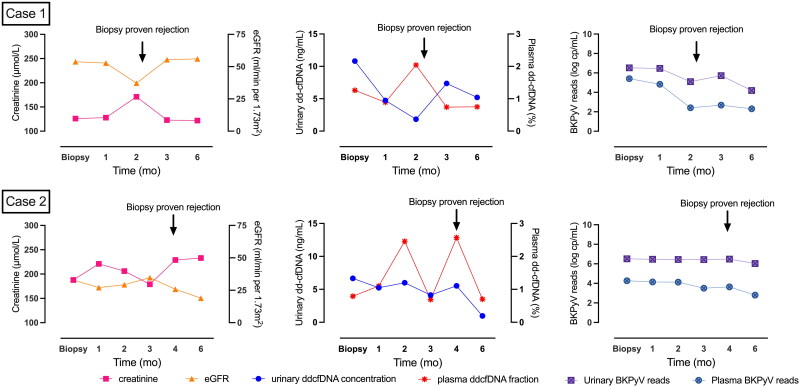
Dynamic surveillance of urinary dd-cfDNA concentrations and plasma dd-cfDNA fractions in two BKPyVAN patients enables early detection of allograft rejection. For the first patient (case 1), increased plasma dd-cfDNA fraction and serum creatinine alongside reductions in both plasma and urine BKPyV reads as well as urinary dd-cfDNA concentration were observed 2 months after the initiation of reduced immunosuppression therapy. Resuming immunosuppression was considered cautiously due to potential rejection events. The subsequent biopsy-proven TCMR IA rejection accompanied with g1 and ptc3 while negative SV40 staining confirmed the previous hypothesis. The patient received pulse methylprednisolone 320 mg per day for 3 days immediately. The decline in plasma dd-cfDNA fraction and serum creatinine afterward indicated complete remission. A slight elevation in urinary dd-cfDNA concentration was observed, and it remained stable after that. In the case of the second patient (Case 2), six assessments of urinary and plasma dd-cfDNA levels were conducted. An elevation in plasma dd-cfDNA fractions occurred in the second month. In the meantime, plasma and urine BKPyV reads remained stable or continued to decrease. Therefore, enhanced immunosuppression was initiated (conversion of ciclosporin + leflunomide + prednisone to tacrolimus + leflunomide + prednisone) with reduced plasma dd-cfDNA fraction and serum creatinine afterward. In the fourth month of follow-up, the patient underwent elevated serum creatinine level with an additional dd-cfDNA test, revealing an increased plasma dd-cfDNA fraction for the second time. A subsequent biopsy-confirmed TCMR IIA rejection accompanied by persistent SV40 positive staining. Despite the positive SV40 staining, the Banff criteria (g1, ptc3, and v1) suggested rejection, particularly humoral rejection. Thus, pulse methylprednisolone 280 mg per day for 3 days of therapy was administered, leading to a successive decline in both urinary and plasma dd-cfDNA levels. However, the allograft function did not show significant recovery.

Recent studies have reported the high risks of allograft rejection in BKPyVAN attributed to either viral-specific immunity or insufficient immunosuppression [1,18,19]. It is crucial to identify indicators that represent the optimal timing for resuming immunosuppression during BKPyVAN to prevent rejection events. In the present study, two patients in CISR cohort experienced biopsy-proven acute rejection after standard BKPyVAN treatment. As presented in Figure 4, both patients had received reduced immunosuppression at the time of biopsy-proven BKPyVAN. The reductions in urinary concentrations observed 2 months after treatment demonstrated effective therapeutic responses. Elevations in plasma dd-cfDNA were noted either concurrently with or before biopsy-proven allograft rejection, serving as a strong indicator to reinforce the maintenance of immunosuppression. These results demonstrated the significance of combining dynamic urinary and plasma dd-cfDNA surveillance to optimize treatment strategies to avoid acute rejection.

### dd-cfDNA levels associated with histopathology manifestations in BKPyVAN

The Banff histopathologic lesions in the biopsy tissues of the BKPyVAN patients were scored by experienced experts. The urinary dd-cfDNA concentrations were significantly elevated in BKPyVAN class 3 (Supplementary Figure 3). Also, we observed that interstitial and tubular injuries were predominant in BKPyVAN. Banff histopathologic lesions, including interstitial inflammation (median i score 2, IQR: 1–2.25), tubulitis (median t score 2, IQR: 1–3), interstitial fibrosis (median ci score 1.5, IQR: 1–2), tubular atrophy (median ct score 2, IQR: 1–2), inflammation in the area of interstitial fibrosis and tubular atrophy (median i-IFTA score 2, IQR: 1–2), and tubulitis in the area of IFTA (median t-IFTA score 1.5, IQR: 0–2) were found in those with BKPyVAN (Supplementary Table 1). The median total inflammation (ti) score was 2, and the median pvl score was 3 among BKPyVAN patients. Banff histopathologic lesion elements associated with antibody-mediated rejection, including glomerulitis (g), peritubular capillaritis (ptc), and C4d, were found to be scarce in BKPyVAN.

**Figure 5.**
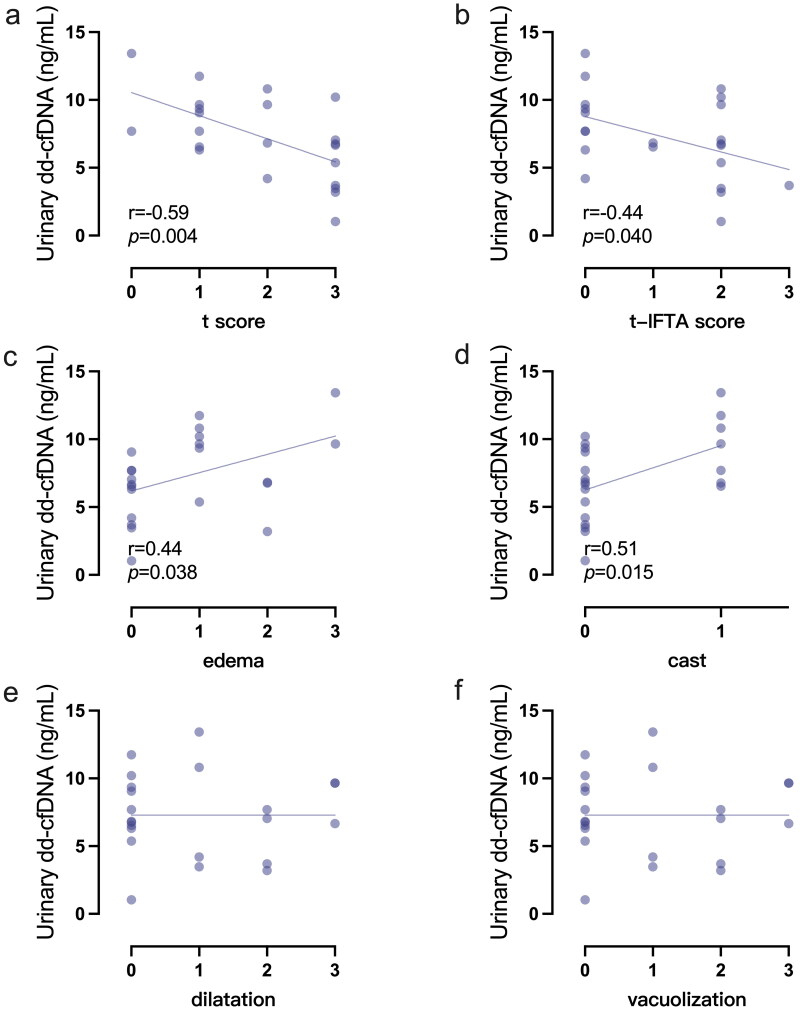
Linear correlations between urinary dd-cfDNA concentrations and Banff histologic elements in BKPyVAN recipients. t score (a) and t-IFTA score (b) were negatively correlated with urinary dd-cfDNA concentrations, while edema (c) and cast (d) were positively correlated with urinary dd-cfDNA concentrations. Dilatation (e), and vacuolization is not significantly correlated with urinary dd-cfDNA concentrations. Bivariate correlations were calculated using Spearman’s coefficient. dd-cfDNA, donor-derived cell-free DNA; r, correlation coefficient.

To further explore the associations between urinary dd-cfDNA levels and histopathological characteristics in BKPyVAN, Pearson’s correlation analysis was performed to evaluate the relationship between urinary dd-cfDNA levels at allograft biopsy and Banff histopathologic lesion scores. Urinary dd-cfDNA concentrations were negatively correlated with tubulitis (r = −0.59, and *p* = 0.004, Figure 5a) and tubulitis in IFTA (r = −0.44, and *p* = 0.040, Figure 5b). Tubular injury is a typical histopathological manifestation in BKPyVAN, characterized by edema, casts, dilatation, and vacuolization [20]. Our results demonstrated that edema (*r* = 0.44, and *p* = 0.038, Figure 5c) and cast (*r* = 0.51, and *p* = 0.015, Figure 5d) were positively correlated with elevation in urinary dd-cfDNA concentrations. However, no significance was observed in dilatation and vacuolization (Figures 5e-f).

## Discussion

In this study, we reported, to the best of our knowledge, the first dynamic surveillance of plasma and urinary dd-cfDNA levels during BKPyVAN treatment in kidney transplant recipients. Our results conveyed four important messages: 1. Reductions in urinary dd-cfDNA concentrations were observed within 2 months after BKPyVAN treatment. 2. For BKPyVAN patients with low urinary dd-cfDNA concentrations, immunosuppression reduction should be cautious due to increased rejection risk. 3. The case series emphasizes the importance of combined plasma and urinary dd-cfDNA monitoring in BKPyVAN to evaluate rejection risk. 4. Tubular injuries associated with urinary dd-cfDNA concentrations in BKPyVAN.

To date, the primary approach for evaluating the therapeutic efficacy of BKPyVAN in clinical practice involves continuous monitoring of BKPyV viremia and viruria after treatment, aiming for consistently undetectable BKPyV viremia [21]. However, this method has certain drawbacks. First, the sensitivity of the real-time polymerase chain reaction (PCR) method for detecting BKPyV loads is relatively low. In our previous study, approximately 30% of biopsy-proven BKPyVAN cases showed negative viremia [14]. Consistent with our findings, the studies of Agrawal et al. [22] and Gras et al. [23] also reported the specific rates of negative viremia in biopsy-proven BKPyVAN. To address this limitation, we applied next-generation sequencing (NGS) in the present study to improve both the specificity and sensitivity of BKPyV detection. Second, BKPyV is latently infected in uroepithelial cells, including ureteral epithelial cells and renal tubular epithelial cells [24]. Positive urine BKPyV reads cannot discern the site of BKPyV infection. By distinguishing cfDNA from donor and recipient sources, dd-cfDNA could identify the location of the injury.

Previous studies have reported that increased urinary dd-cfDNA concentrations are related to the diagnosis of BKPyVAN with a cutoff value of approximately 6–8 ng/mL [14,15]. Our previous study showed that increased urinary dd-cfDNA concentration, rather than plasma dd-cfDNA fraction, was observed in BKPyVAN, with a cutoff value of 7.81 ng/mL to distinguish BKPyVAN from TCMR [14]. Consistent with our findings, Chen et al. also uncovered specific elevations in urinary dd-cfDNA concentrations in BKPyVAN (median level, 12.22 ng/mL) [13]. And in another study, higher urinary dd-cfDNA levels were observed in proved and probable BKPyVAN compared with possible and resolving BKPyVAN [15]. The optimal surveillance schedule, concerning both timing and frequency of measurements, remains undetermined [25]. The present study was conducted to address this gap. It is widely believed that persistent BKPyV viruria for 2–6 weeks could progress to BK viremia, and again, persistent BKPyV viremia for another 2–6 weeks could eventually develop into BKPyVAN [26–28]. A study detailing continuous biopsies in 61 BKPyVAN patients revealed that the median time for SV40 remission on histopathology was 5 months, with most patients achieving persistent serum negativity [5]. Additionally, BKPyV viremia significantly declined at 3 months after effective therapy. Consequently, the follow-up time points selected in the present investigation for dd-cfDNA surveillance were 1, 2, 3, and 6 months after biopsy-proven BKPyVAN to reveal the disease remission process based on the duration of BKPyV viremia and viruria. Although the current study lacks a control group with stable allograft function due to unmatched follow-up duration, our prior findings [14] demonstrated a median urinary dd-cfDNA concentration of 4.4 ng/mL (*n* = 12) in such patients. These results underscore the critical importance of dd-cfDNA surveillance during the initial 3 months in BKPyVAN. Nevertheless, further large-scale, longitudinal studies are warranted to elucidate the long-term utility of dd-cfDNA monitoring in BKPyVAN management.

Our study revealed, dynamic changes in plasma and urinary dd-cfDNA levels following immunosuppression reduction treatment in BKPyVAN patients. Notably, a reduction in urinary dd-cfDNA concentration was observed after the initiation of standard BKPyVAN treatment, indicating a more rapid therapeutic response compared to plasma and urinary BKPyV reads. The decline in urinary dd-cfDNA concentrations was evident as early as 2 months post-treatment and remained steady at the 3- and 6-month follow-ups. The majority of patients (18/20) in the CISR cohort showed a decline in urinary dd-cfDNA concentration by the second month. Although two patients experienced increased urinary dd-cfDNA levels, these elevations remained within an acceptable range. The response rate of BKPyVAN withdrawal immunosuppressive therapy at 2 months based on urinary dd-cfDNA concentration was as high as 84%. Our findings highlighted the importance of urinary dd-cfDNA concentration in determining disease remission and obviating the need for invasive repeated biopsy.

Attention should be paid to whether the preceding immunosuppression reduction was initiated before the biopsy. While positive SV40 staining in allograft tissue indicates BKPyV infection, it does not differentiate between disease onset, significant histopathological damage, or residual virus during regression. Consequently, making clinical decisions about reducing or resuming immunosuppressants based solely on SV40 staining is challenging. In our study, BKPyVAN patients with preceding immunosuppression reduction (EISR cohort) demonstrated lower urinary dd-cfDNA concentrations of 3.69 ng/mL, which is close to the urinary dd-cfDNA concentrations observed in patients with stable function (4.4 ng/mL). As a result, their immunosuppressants were maintained intact. Comparisons between CISR and EISR cohorts revealed differences in urinary dd-cfDNA concentrations during BKPyVAN remission. These results highlight the ability of urinary dd-cfDNA levels to differentiate the onset and different stages of BKPyVAN, thereby avoiding over-reduction of immunosuppressants. While surveillance of BKPyV viremia and viruria has been a classic process in monitoring BKPyVAN for disease remission [29–31], no significant difference in BKPyV viremia and viruria was observed between CISR and EISR cohorts.

The measurement of plasma dd-cfDNA% is widely accepted as a noninvasive biomarker to indicate acute rejection with a cutoff value approximately 0.74%–1.0%. Plasma dd-cfDNA% less than 0.5% represents allograft stable status in the ADMIRAL study [32]. It is revealed by the DART study [9] that median dd-cfDNA% levels in ABMR, TCMR IB, and IA were 2.9%, 1.2%, and 0.2%, respectively. Elevated dd-cfDNA levels higher than 1% is a strong evidence of concurrent acute rejection, especially ABMR, which is consistent with the findings in the Trifecta Study that dd-cfDNA% correlated with all 20 top probe sets associates with ABMR and all types of rejection [7]. Sigdel et al. showed that recipients with BK viremia correlated with increased plasma dd-cfDNA% in 4 patients [33]. A recent large-scale investigation demonstrated that increased dd-cfDNA% levels in rejection events including mixed rejection, acute TCMR, active ABMR, and chronic active ABMR [34]. The elevations in plasma dd-cfDNA% is a strong indicator of allograft rejection.

The prevalence of acute rejection in BKPyVAN is significantly elevated due to either insufficient immunosuppression or activation of BKPyV-specific immunity [35]. Patients with BKPyV infection face a 2.5-fold increase in the risk of death-censored graft loss [1]. The complex interplay between BKPyV and allograft rejection poses a challenge in determining the optimal degree of immunosuppression reduction to interrupt viral replication while balancing the risk of inducing allograft rejection. Notably, two patients experienced acute rejection during immunosuppression reduction with histopathological findings of TCMR IA and IIA, respectively. Dynamic monitoring of both urinary and plasma dd-cfDNA levels showed antecedent plasma dd-cfDNA fraction increases in these patients, indicating an early sign of rejection. Another patient also experienced an increase in plasma dd-cfDNA fraction with subsequent allograft biopsy confirmed the diagnosis of borderline changes with persistent SV40 positive staining. Enhanced immunosuppression therapy (conversion of ciclosporin + leflunomide + prednisone to tacrolimus + mycophenolate + prednisone) was applied considering about stable serum creatinine. Reductions in plasma dd-cfDNA fraction and stable urinary dd-cfDNA concentrations indicated complete remission of rejection. Urinary dd-cfDNA concentrations contributed to determining the successful clearance of BKPyVAN. In contrast, plasma dd-cfDNA fractions helped in deciding the timing for returning to routine maintenance immunosuppression to counteract rejection. We advocate for the use of both urinary and plasma dd-cfDNA measurements to inform personalized treatment decisions for BKPyVAN and to monitor immunologic risk for rejection.

Our study revealed a significant positive correlation between urinary dd-cfDNA concentrations and specific tubular injury patterns (edema and cast) in BKPyVAN, whereas no such association was observed with vacuolization or dilatation. After brush border loss and non-isometric cytoplasmic vacuolization, tubular cells undergo progressive degeneration characterized by either cytoplasmic fragmentation or cytoplasmic fragmentation or detach entirely into the lumen, ultimately forming obstructive casts. Concurrently, tubular dilatation occurs as a consequence of both increased intraluminal pressure and cytoskeletal alterations. The combined effects of tubular obstruction and inflammatory processes facilitate transepithelial fluid leakage across denuded basement membranes, resulting in progressive interstitial edema formation. These pathological changes were quantitatively analyzed in the study by Pieters et al. [20], which demonstrated a significant inverse correlation between the severity of interstitial edema/cast formation and renal function. Together with our findings, these data demonstrate that edema and cast formation reflect more advanced stages of tubular injury compared to vacuolization and dilatation. This progression leads to more extensive tubular cell death, which mechanistically explains the observed elevation in urinary dd-cfDNA concentration during allograft injury.

There are several limitations in this study. First, the sample size was small. Due to the relatively low prevalence of BKPyVAN in our center, only 25 BKPyVAN cases were enrolled, especially in EISR cohort (only 5 cases) which might raise concerns about statistical power and generalizability of findings. Second, allograft injuries, including ABMR, TCMR, borderline changes, IgAN, and IFTA were excluded from this study. The primary goal is to assess the dynamics of urinary and plasma dd-cfDNA levels during BKPyVAN treatment, their association with allograft injury, and their potential to identify the risk of acute rejection. Further research is needed to address this gap. Third, protocol and continuous biopsies were not available, resulting in a lack of histopathological evidence for disease onset and remission.

Accordingly, we combined serum creatinine levels with plasma and urinary BKPyV reads as indicators of disease remission. Forth, the sample storage conditions (4–37 °C for less than 24 h) may introduce variability in cfDNA degradation. Larger-scale clinical trials are warranted to evaluate the clinical utility of dynamic plasma and urinary monitoring of dd-cfDNA.

Overall, it is the first dedicated study to explore the value of dynamic dd-cfDNA surveillance in BKPyVAN post-kidney transplantation. Our findings reveal a noteworthy reduction in urinary dd-cfDNA concentrations following the initiation of immunosuppression reduction. The concurrent assessment of plasma dd-cfDNA is crucial for monitoring acute rejection. We recommend the routine measurement of both urinary and plasma dd-cfDNA at 2 months after immunosuppression reduction therapy to assess therapeutic response and potential rejection risk. The combined use of urinary and plasma dd-cfDNA offers valuable guidance for personalized treatment decisions in BKPyVAN management.

## Supplementary Material

Supplementary materials.docx

## Data Availability

The original data during the current study are available from the corresponding author on reasonable request.
