# Supplementary material for: Urinary and plasma donor-derived cell-free DNA for noninvasive monitoring of BK polyomavirus-associated nephropathy in kidney transplant recipients: a prospective cohort study
Source: Ren Fail. 2025 Jun 25;47(1):2521452. doi: 10.1080/0886022X.2025.2521452 (PMC12893493; doi:10.1080/0886022X.2025.2521452)
Supplement: Supplementary materials.docx [file IRNF_A_2521452_SM2474.docx]

**Supplementary materials**

**Title: Urinary and Plasma Donor-Derived Cell-Free DNA for Non-Invasive Monitoring of BK Polyomavirus-Associated Nephropathy in Kidney Transplant Recipients: A Prospective Cohort Study**

Luying Guo, Sulin Luo, Rongfang Shen, Pengpeng Yan, Meifang Wang, Tianlu Zhang, Junhao Lv, Guangjun Liu, Hongfeng Huang, Zhimin Chen, Huiping Wang, Wenhan Peng, Jiangyong Wu, Jianghua Chen, Rending Wang

**Supplementary Figure 1. BKPyV subtypes in kidney transplant recipients.** The majority of BKPyV genome variants were BKPyV-Ic and IVc-1 in our study.

**Supplementary Figure 2. Serum creatinine (a) and eGFR (b) levels in CISR and EISR cohort at Biopsy, 1, 2, 3, and 6 months afterward.**


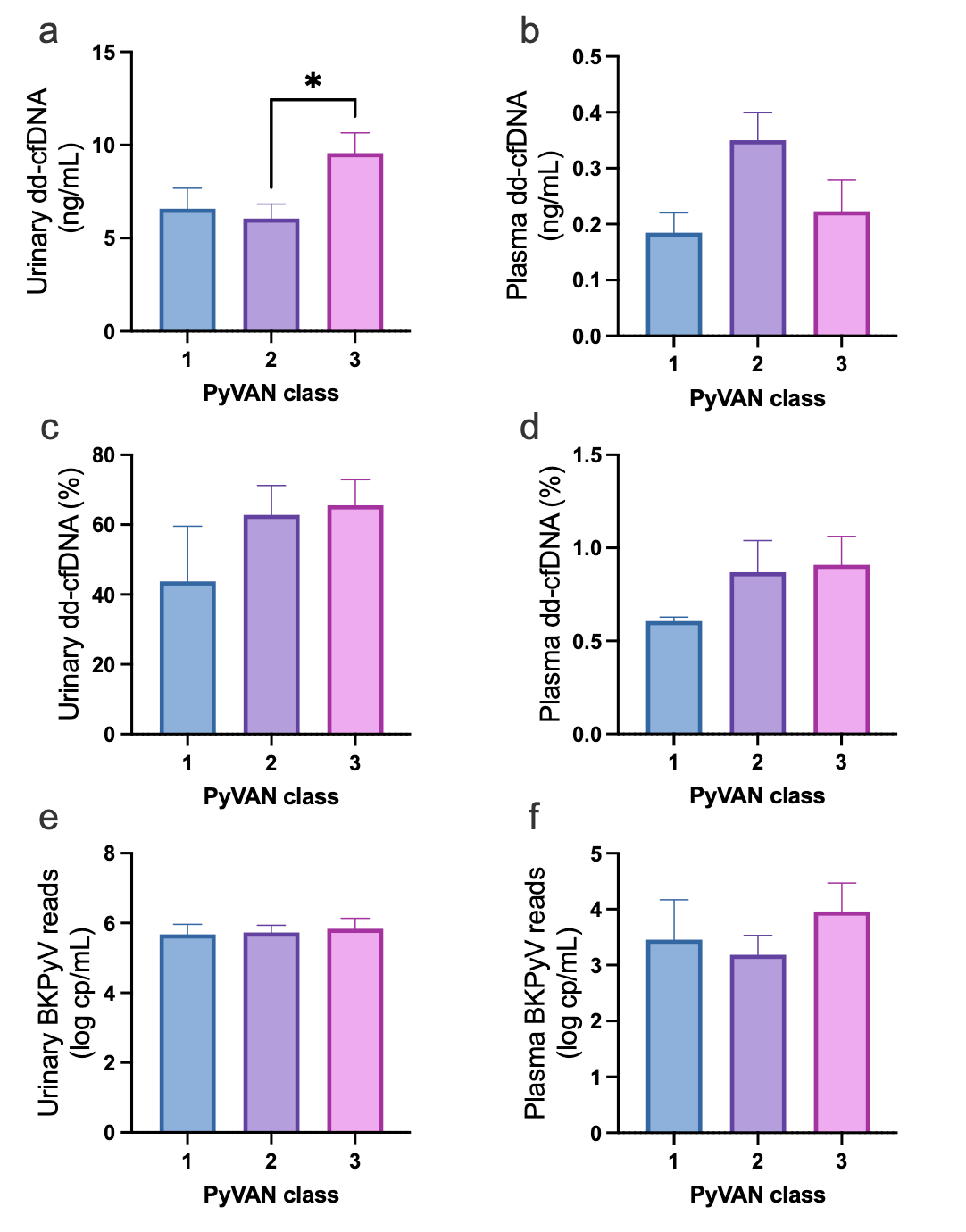


**Supplementary Figure 3. Correlations between dd-cfDNA levels and PyVAN classes.**

**Urinary and plasma dd-cfDNA concentration (a and b), urinary** **and plasma dd-cfDNA fraction (c and d),** **urinary and plasma BKPyV reads (e and f). Mean±SE, *, p < 0.05**

**Supplementary table 1. Banff histopathologic score in allograft biopsy of BKPyVAN patients.**

| Elements | Banff histopathologic score Median (Quantile) |
| --- | --- |
| i | 2 (1-2.25) |
| t | 2 (1-3) |
| v | 0 (0-0) |
| g | 0 (0-0) |
| ptc | 0 (0-2) |
| C4d | 0 (0-0) |
| ci | 1.5 (1-2) |
| ct | 2 (1-2) |
| cv | 0 (0-2) |
| ptcml | 0 (0-0) |
| ti | 2 (1-2.25) |
| i-IFTA | 2 (1-2) |
| t-IFTA | 1.5 (0-2) |
| pvl | 3 (1-3) |
